# Commentary: Rewriting the Valuation and Salience of Alcohol-Related Stimuli *via* Memory Reconsolidation

**DOI:** 10.3389/fpsyt.2016.00041

**Published:** 2016-03-17

**Authors:** Udi E. Ghitza

**Affiliations:** ^1^Center for the Clinical Trials Network, National Institute on Drug Abuse, National Institutes of Health, Bethesda, MD, USA

**Keywords:** alcohol drinking, memory retrieval, alcohol use disorder, drug use disorder, substance use disorder, addiction, alcohol abuse, drug abuse

Neuroadaptations of neuronal ensembles in the mesocorticolimbic reward system mediate memory processes, which link drug-seeking experiences to the contexts in which they occur (1–3). In many individuals with alcohol or drug-related problems, such memory processes imbue drug-related contexts with motivational salience – a resonant incentive motivational attribution attached to drug-predictive cues, which drives addictive behaviors (4–6). When such memories are recalled, they are thought to return to a volatile state in which they could be altered before undergoing reconsolidation for future use (7–9). A recent notable study sheds light on a condition by which to block memory reconsolidation and subsequent responsivity to alcohol-related stimuli in individuals with alcohol-related problems (10).

In hazardous drinkers, Das et al. (10) destabilized alcohol-related memory reconsolidation through a procedure which maximized omission prediction error (PE) through explicitly guided expectancy violation during memory retrieval (10). PE involved these drinkers being led to believe that they will be able to drink alcohol through presentation of alcohol conditioned stimuli to reactivate their alcohol-related memories and explicit instructions designed to maximize the expectation of alcohol reward but then were unexpectedly prevented from drinking. Ten minutes later, alcohol-specific cues were paired with disgusting tastes and images in a counter-conditioning procedure designed to rewrite the alcohol-related memory. Hazardous drinkers exhibited reduced attentional bias to and liking of alcohol-related stimuli and a reduced positive expectancy of alcohol 1 week following this PE and counter-conditioning procedure. Interestingly, this memory reactivation-dependent reduction in the motivational salience and valuation of alcohol-related stimuli generalized to novel alcohol cues. Two comparison groups of hazardous drinkers were included – one that did not undergo PE upon alcohol-related memory retrieval and one in which the alcohol-related memory was not retrieved. Neither of these comparison groups exhibited this reduction in motivational salience or valuation of alcohol-related stimuli, relative to neutral stimuli. This study suggests an innovative approach to destabilize retrieval of alcohol-related memories using a PE manipulation paired with counter conditioning. This method could be useful to disrupt or weaken maladaptive alcohol memories in clinical practice if future research shows it to be a selective alcohol memory-rewriting intervention.

Further research should identify the necessary and sufficient parameters for producing durable weakening of alcohol- and drug-related memories by fortifying PE-induced memory destabilization, together with medication-assisted therapy (MAT) and counter conditioning. A fuller insight gleaned from understanding what is required for memory reconsolidation to be blocked in a long-lasting manner should aid development of treatments to disrupt memories associated with substance use disorders (SUDs). First, it would be helpful for research to test how efficacy of PE-based strategies to block memory reconsolidation could be enhanced by pharmacological adjuncts, for example, adrenergic beta-receptor blockers or adrenergic alpha-2 receptor agonists, which prior research suggests could be useful aids to impair memory reconsolidation (11, 12). Second, research is needed to identify the parameters necessary to optimize the efficacy of counter conditioning and to extend the applicability of the Das et al. (10) memory-destabilization approach to treating other SUDs. Further research should also identify how to maximize the generalizability of memory alteration to other drug-predictive contexts, in order to generalize this relearning across various drug-related surroundings.

Individuals with multiple SUDs may require more intense care to augment any beneficial effects of rewriting maladaptive alcohol- or drug-related memories. Thus, precision medicine research also needs to be conducted to establish effectiveness of stepped care, tailored treatment approaches using nuanced clinical decision support (CDS) tools, and shared decision-making approaches to move SUDs patients into more intensive interventions, which attend to their personalized phenotypes that present obstacles to their recovery and overall wellness, their risk concerns, and preferences. Research is also needed on how to optimize shared decision making between patients and providers to tailor personalized medical care to particular problems and risk categories which individual patients report as most distressing to their functioning in daily lives. Thus, ultimately, precision medicine research will also need to assess the effectiveness of patient-centered care approaches utilizing shared decision making to treating substance use problems and co-occurring behavioral health issues, considering individual preferences for treatment options, taking into account: for instance, (a) SUD severity, (b) readiness of patients to change their harmful substance use, (c) underlying reasons for readiness or hesitancy to do so, (d) presence of co-occurring psychiatric and other medical conditions, and (e) how to help patients in instituting a patient-centered treatment plan consistent with their co-occurring conditions, readiness to change, and their preferences/values (13, 14).

Clinical research programs with practice-based research networks serving patient populations with substance use problems can play an important role in this endeavor. Existing health information system infrastructures in practice-based research networks should be leveraged to help accelerate this line of research, which, in turn, could lead to more efficient enrollment of patients into trials, accelerated recruitment of providers to deliver study interventions, and more efficient use of existing health information technologies such as electronic health record systems (EHRs) (13, 14).

## Author Contributions

UG, Ph.D., undertook a review of the literature, conceived of this general commentary, and wrote and reviewed all drafts.

## Conflict of Interest Statement

The author declares that the research was conducted in the absence of any commercial or financial relationships that could be construed as a potential conflict of interest.
